# Super‐resolution microscopy as a potential approach to diagnosis of platelet granule disorders

**DOI:** 10.1111/jth.13269

**Published:** 2016-03-17

**Authors:** D. Westmoreland, M. Shaw, W. Grimes, D. J. Metcalf, J. J. Burden, K. Gomez, A. E. Knight, D. F. Cutler

**Affiliations:** ^1^ MRC Laboratory for Molecular Cell BiologyUniversity College LondonLondonUK; ^2^ Endothelial Cell Biology LaboratoryLondonUK; ^3^ Analytical Science DivisionNational Physical Laboratory, TeddingtonMiddlesexLondonUK; ^4^ Imaging Informatics DivisionBioinformatics InstituteSingaporeSingapore; ^5^ Nikon InstrumentsLondonUK; ^6^ Electron Microscopy LaboratoryLondonUK; ^7^ Katherine Dormandy Haemophilia Centre and Thrombosis UnitRoyal Free London NHS Foundation TrustLondonUK

**Keywords:** diagnostic imaging, Hermansky‐Pudlak syndrome, platelet storage pool disorder, platelets, super‐resolution, structured illumination microscopy

## Abstract

**Essentials**

- Deficiencies in size, number or shape of platelet granules are associated with bleeding symptoms.
- Super‐resolution microscopy (SRM) facilitates the diagnosis of structural platelet disorders.
- SRM can deliver quantitative, automated, unbiased high‐throughput morphometric analyses.
- Using CD63 as a marker, Hermansky‐Pudlak patients are easily distinguished from controls.

**Summary:**

**Background**

Many platelet functions are dependent on bioactive molecules released from their granules. Deficiencies of these granules in number, shape or content are associated with bleeding. The small size of these granules is such that imaging them for diagnosis has traditionally required electron microscopy. However, recently developed super‐resolution microscopes provide sufficient spatial resolution to effectively image platelet granules. When combined with automated image analysis, these methods provide a quantitative, unbiased, rapidly acquired dataset that can readily and reliably reveal differences in platelet granules between individuals.

**Objective**

To demonstrate the ability of structured illumination microscopy (SIM) to efficiently differentiate between healthy volunteers and three patients with Hermansky‐Pudlak syndrome.

**Methods**

Blood samples were taken from three patients with Hermansky‐Pudlak syndrome and seven controls. Patients 1–3 have gene defects in *HPS1*,*HPS6* and *HPS5*, respectively; all controls were healthy volunteers. Platelet‐rich plasma was isolated from blood and the platelets fixed, stained for CD63 and processed for analysis by immunofluorescence microscopy, using a custom‐built SIM microscope.

**Results**

SIM can successfully resolve CD63‐positive structures in fixed platelets. A determination of the number of CD63‐positive structures per platelet allowed us to conclude that each patient was significantly different from all of the controls with 99% confidence.

**Conclusions**

A super‐resolution imaging approach is effective and rapid in objectively differentiating between patients with a platelet bleeding disorder and healthy volunteers. CD63 is a useful marker for predicting Hermansky‐Pudlak syndrome and could be used in the diagnosis of patients suspected of other platelet granule disorders.

## Introduction

**Figure 1:**
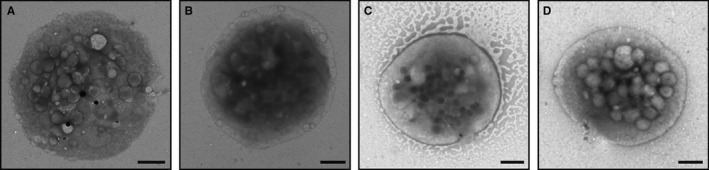
Whole mount EM of Platelets. Analysis of dense granules by whole mount EM may be regarded as the “gold standard” for determining numbers of these organelles per platelet. Examples of single platelets from one of the 7 healthy volunteers (A) and from each HPS Patient 1, 2 and 3 (B,C,D) used in this study are shown. Note the lack of dense granules (black spots) seen in the HPS patients. The criteria for counting dense granules are described in Supplementary Figure 1. Scale bars: 1 μm.

Platelets are cell fragments derived from megakaryocytes. Their distinguishing features include an open canalicular system, a dense tubular system (smooth endoplasmic reticulum) and secretory organelles. Historically, their granules have been classified into distinct groups based on their content and appearance by electron microscopy (EM): dense granules, α granules, multivesicular bodies and lysosomes 1, plus now also T granules 2. Most platelet functions are mediated via the release of bioactive molecules from these organelles. The critical role of platelets in secondary hemostasis is therefore dependent on their integrity. Deficiencies of these organelles, including abnormalities in granule number or size, are associated with bleeding symptoms 3. In genetic disorders such as Hermansky‐Pudlak syndrome (HPS) and Chediak‐Higashi syndrome (CHS) the absence of dense granules significantly slows the rate of effective hemostasis at the site of an injury 4. Dense granules (also known as dense bodies or delta granules) can be visualized using EM and are recognizable by the presence of a very electron‐dense core (Fig. 1, Figure S1), which is caused by their high calcium content. They also contain serotonin, histamine, ADP, ATP, GDP, GTP, magnesium pyrophosphate 5 and polyphosphate 6. Platelets are thought to each contain three to eight 7 of these organelles.

The established tools available to clinicians for diagnosing dense‐granule disorders are currently limited. Discussion within the clinical community suggests that there is room for additional new techniques, for example as in recent reports 8, 9 suggesting the further use of fluorescence‐activated cell sorting (FACS)‐based assays as a diagnostic tool in this context. Currently, assays for a potential dense‐granule disorder include aggregation in response to a panel of agonists, measurement of platelet nucleotides and/or ATP release and direct counting of dense granules using EM.

Platelet aggregation is measured by light transmission aggregometry (LTA) and using platelet agonists, such as arachidonic acid, thrombin, ADP, epinephrine, collagen and ristocetin 4, can differentiate between controls and patients. Despite its widespread use, there are caveats regarding LTA results: the measurements are not strictly comparable to physiological platelet aggregation as the separated platelets in platelet‐rich plasma (PRP) only form aggregates following the addition of soluble agonists under low shear conditions (stirring). LTA tells us little about structural malformations. Nucleotide or ATP release measurements also suffer from the latter defect.

Some structural information about dense granules can be obtained using a whole‐mount EM technique 10, 11. An absence or reduction of dense granules, due to mutations in the cellular machinery used in granule formation, is found in patients with genetic disorders such as HPS and CHS 10. Using whole‐mount EM to resolve platelet granules is currently the reference standard for diagnosing a dense‐granule defect. However, quantitative EM analysis requires an electron microscope and specialized skills, is time consuming and is very difficult to automate. Without markers, granules are difficult to define and identification will differ depending on the analyst (12, Figure S1). Finally, the equipment and specialized techniques required for both of these analyses are not available in most hospitals, requiring patients with platelet disorders to travel long distances to a specialized center.

The limitations of these current techniques suggest an unmet need for new approaches to diagnosing platelet granule disorders (PGDs). Until the recent development of super resolution microscopy (SRM) 13, 14, 15, one promising alternative approach, the acquisition of quantitative data from immuno‐histochemical staining has not been effective in platelets. Platelets are 2–5 μm in diameter; their dense granules are approximately 150 nm and their alpha granules 200–400 nm 16. While confocal microscopy typically offers a lateral spatial resolution of 200–300 nm, the new SRM methods, using a variety of different strategies to overcome the diffraction limit, can resolve structures in the 10–200 nm range, and thus can easily resolve individual platelet granules. This improved spatial resolution supports accurate visualization of distinct objects within a platelet that are positive for a specific marker. If SRM is coupled with current automated image analysis methods, quantitative data regarding the platelet granules can be extracted.

Here we provide a proof‐of‐principle dataset showing the ability of one SRM approach, structured illumination microscopy (SIM 17), to discriminate between a group of healthy controls and three patients with platelet storage disorders. In this limited analysis, the dense‐granule abnormalities caused by HPS were investigated using CD63 as a marker. This particular marker was used because of reports of changes to CD63 in HPS patients 18. Our results suggest that this general approach could be a valuable addition to the diagnosis of patients with platelet storage disorders, not least because it would be widely applicable once a panel of antibodies that would allow detection of changes to all the platelet organelles had been validated.

## Methods

### Patient phenotyping

The three patients with HPS had a similar clinical phenotype. All were noted to have oculocutaneous albinism from birth and a history of excessive bleeding after trauma. Patient 1 has pulmonary fibrosis but patients 2 and 3 do not. Platelet function assays showed similar results in all three cases with reduced LTA to a standard panel of agonists and very low levels of platelet ADP documented by bioluminescent assay of the nucleotide content in lysed platelets. Genetic analysis was performed by exome sequencing on the ThromboGenomics platform that includes *HPS1‐9*.

Patient 1 had compound heterozygosity for two single nucleotide deletions resulting in frameshifts and introducing premature stop codons in the *HPS1* gene: c.418delG and c.1189delC predicted to result in p.A140Rfs*34 and p.Q397Sfs*1. Patient 2 had compound heterozygosity for two single nucleotide duplications resulting in frameshifts and introducing premature stop codons in the *HPS6* gene: c.902dupC and c.1083dupC predicted to result in p.T303Hfs*64 and p.G362Rfs*5. Patient 3 had homozygosity for a single nucleotide change introducing premature stop codons in the *HPS5* gene: c.2232T>A predicted to result in p.Cys744*. Controls were healthy volunteers. This work was approved by the relevant UK research ethics committee and all participants gave their written informed consent.

### Preparing platelets for analysis

First 7 ml of whole blood was collected into a 1 : 7 solution of acid citrate dextrose (ACD) and centrifuged at 180 × *g* for 17 min. PRP was collected from the supernatant and left for 30 min, then diluted 1 : 10, 1 : 50, 1 : 100 and 1 : 500 in HEPES Tyrode's buffer and fixed with formaldehyde in phosphate buffered saline (PBS) at a final concentration of 4% for a minimum of 10 min before centrifugation at 600 × *g* for 5 min in a Beckman–Coulter Allegra 6R onto poly‐L‐lysine‐coated coverslips and washed once with PBS. Permeabilization with 0.2% TX‐100 in PBS was followed by incubation with primary (antitubulin antibody from Cytoskeleton Inc., (Denver, CO, USA) catalogue number ATN02, used at a concentration of 1 : 200; anti‐CD63 antibody from Abcam (Cambridge, UK), catalogue number AB59479, used at a concentration of 1 : 100) then secondary antibodies (concentration 1 : 500) conjugated to Alexa Fluor dyes (Molecular Probes, Life Technologies, Paisley, UK) or Cy5 (Jackson ImmunoResearch Laboratories, West Grove, PA, USA) before mounting (ProLong Gold antifade reagent, Life Technologies). Imaging was carried out using an inverted wide‐field fluorescence microscope (IX71, Olympus, Tokyo, Japan) modified for SIM, as previously described 19, 20.

Once the blood was taken, the samples were fixed and stored at 4 °C for up to a month or imaged directly. We found no difference in the quality of images relating to the length of time between collection and imaging (data not shown), strongly suggesting that distant collection and fixation of samples for centralized processing should be possible.

### Super‐resolution microscopy of platelets

Each SRM image was reconstructed from a sequence of raw images of the sample acquired under excitation with nine different sinusoidal illumination patterns, as previously described 12, with a typical exposure time of 200 ms. For comparison, diffraction‐limited images were created by summing all nine raw images. out‐of‐focus light in each image was suppressed by using linear weighting of Fourier space image information components, as described previously 19, 20. Two‐color images were acquired sequentially under excitation of the sample with laser light at 488 nm (CD63) and 561 nm (tubulin). Image *z*‐stacks were obtained by axially translating the specimen in 0.2‐μm steps using a piezoelectric translation stage (NanoScanZ, Prior Scientific, Cambridge, UK).

### Automated image analysis workflow

**Figure 2:**
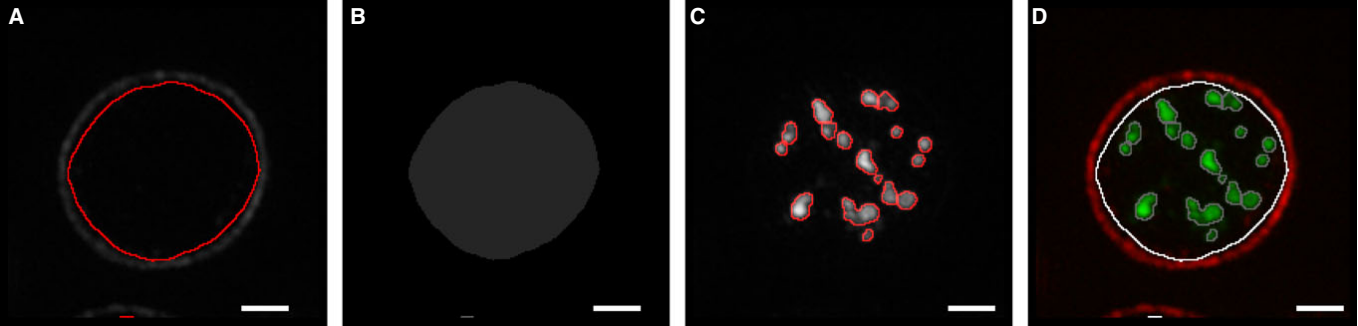
Structured illumination microscopy (SIM) segmentation workflow. SIM images were analyzed as follows: platelets were segmented using the image of the tubulin and corresponding CD63 image (A) and then filled (B). Platelets were distinguished from debris by their area and circularity. Granules within the filled area (B) were segmented according to a threshold value obtained from ‘Moment‐preserving thresholding’ (C). The platelet in which each CD63 granule is located was recorded (D) and morphometric features (including area) of each granule were measured. Scale bars: 1 μm.

An automated workflow written as an ImageJ macro was used to segment SIM images of CD63 granules and assign granules to their constituent platelets (Fig. 2). Platelets were segmented using the addition of the tubulin image to its corresponding CD63 image. Uneven background illumination was removed from this composite image using a rolling ball background subtraction with a radius of 20 pixels 21. Image noise was reduced with Gaussian blurring, with a sigma value not impacting the image resolution, and finally a minimum error threshold was applied 22. The segmented tubulin‐labeled structures in the binary image were filled and a watershed transform performed to separate touching platelets. To demarcate the interior of the platelet, the filled area was eroded to avoid any contribution of CD63 found at the platelet surface. Segmented structures with an area of less than 2.0 μm^2^ (the reported diameter of platelets varies from 2 to 5 μm and in this set of individuals our tubulin ring measurements gave areas ranging from 2.3 to 3.1 μm^2^) or circularity (defined as 4π(area/perimeter^2^) less than 0.7) were removed 23; for the remaining foreground objects, morphometric measurements were taken.

Segmentation of CD63‐positive structures for each of the seven control CD63 image datasets was performed according to a threshold value obtained from moment preserving thresholding 24. The three patient data threshold values were set according to control data that were acquired on the same day. Segmented CD63 granules that were overlapping were separated with a watershed transform and areas smaller than 0.01 μm^2^ were considered background and removed (average diameter of dense granules 150 nm, area ≥ 0.018 nm^2^). The platelet in which each CD63 granule was located was saved and morphometric features (including area) were measured.

### CD63 distribution measurements

From a total population of 2812, platelets were selected with a more stringent circularity criterion of > 0.85, giving a reduced sample of the 1549 most circular platelets. To assess the spatial distribution of CD63 within each platelet, the radial distribution of pixel values in both the CD63 and tubulin channels was computed using the radial profile plugin in ImageJ 25. The plugin creates a set of concentric rings from the center of each platelet to the edge and in each ring calculates the normalized integrated density, which is the sum of pixel values in that ring divided by the number of pixels in the ring. The edge of the platelet is defined as the radial distance to the ring with maximum normalized integrated density in the tubulin channel. The radial distance is normalized for each platelet by division of the radial distance from the center for each ring by the distance to the edge ring. Similarly, radial integrated density is normalized by dividing the integrated density for each ring by the maximum integrated density value for that platelet. Normalization of both the radial distance and radial integrated density allows platelet distributions to be compared independently of the size of the platelet and the pixel intensities. In order to compare spatial distributions across different segmented platelets, CD63 channel pixel values were normalized by the total integrated signal and the radial distance from the platelet center was normalized to the maximum radius of the tubulin ring.

### Whole‐mount electron microscopy

PRP was diluted in HEPES Tyrode's buffer and centrifuged at 600 × *g* for 5 min in a Beckman Coulter Allegra 6R onto formvar‐coated mesh copper grids. The grids were washed twice in water, dried for 20 min and imaged directly by TEM without fixation (Tecnai Spirit, FEI, Hillsboro, OR, USA). Images of whole‐mount platelets were randomized and counted by one analyst (Fig. 1). All images were counted in one sitting to avoid variation in counting criteria. For this many samples, it took a relatively experienced cell biologist accustomed to examining all kinds of images around 6 h to count all the dense granules from 1008 platelets for this dataset. Images too ambiguous to count were discarded. An example of which objects were classified as dense granules is provided in Figure S1.

## Results

For this study, we chose to use SIM for its simplicity of sample preparation, compatibility with routinely used fluorescent labels and fast image‐acquisition rates. Platelet samples prepared by a simple robust protocol (Methods) were imaged on a custom‐built SIM and, in parallel, by whole‐mount EM to provide a reference‐standard diagnostic image‐set for comparison.

**Figure 3:**
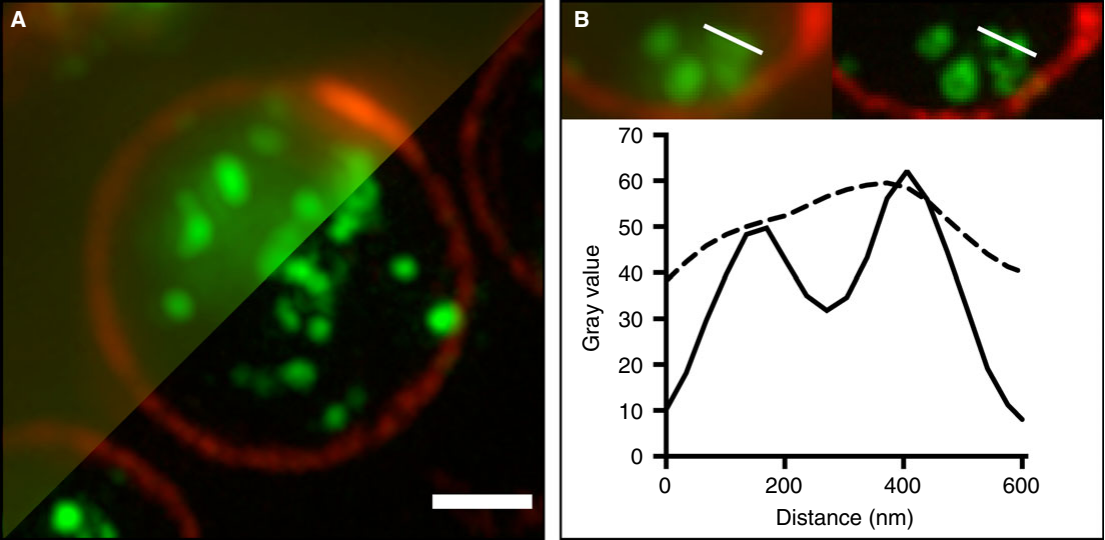
Imaging platelets by SIM. (A) Platelets (prepared as in Methods) were imaged by SIM (lower right) with a diffraction‐limited widefield microscopy image (upper left) shown for comparison. The images show the platelet perimeter (marginal band) labelled (red) with anti‐tubulin (Cytoskeleton Inc., sheep polyclonal ATNO2) and the internal structures (green), largely dense granules, labelled with anti‐CD63 (Abcam, mouse monoclonal AB59479). (B) The improved contrast and resolution in the SIM image (right) is clearly apparent from a 600 nm line profile (lower panel) taken through two closely adjacent structures. This improvement in image quality facilitates granule separation, allowing for an efficient, high throughput, automated, unbiased workflow. Scale bar: 1 μm.

Fixed platelets were centrifuged onto poly‐L‐lysine coverslips to create a high density of platelets to analyze, because this dramatically reduces imaging time. To determine the number of CD63‐positive structures per platelet, we co‐stained for tubulin to demarcate the perimeter of individual platelets, within which segmented granules were counted (Fig. 2). This allowed an analysis based on granules per platelet, thus giving a more sensitive readout. The improved spatial resolution and removal of out‐of‐focus background obtained by SIM is clearly apparent when compared with wide‐field microscopy (Fig. 3A, B). The improved images obtained with SIM greatly facilitated the identification and counting of CD63‐positive structures (Figs. 3B and 2).

**Figure 4:**
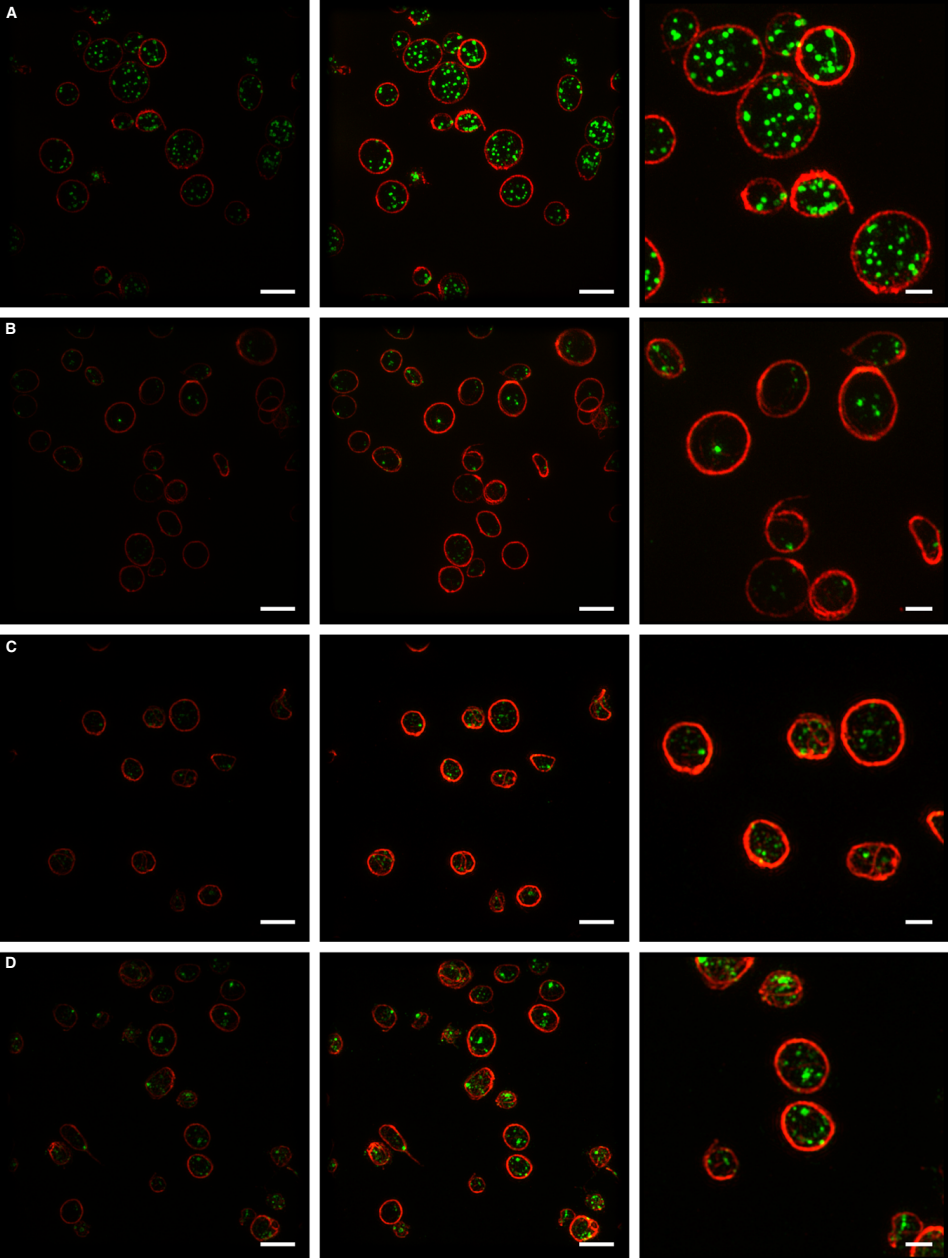
Structured illumination microscopy (SIM) of platelets. Example SIM images of platelets from one of the seven healthy volunteers (A) and from each Hermansky‐Pudlak syndrome (HPS) patient (1, 2 and 3) (B, C, D) used in this study. The images in the first column are an example of the SIM data as analyzed (Fig. 6); images in the second and third columns have been similarly contrast enhanced, with a magnified portion of those images shown in column 3. The two‐color images were acquired sequentially under excitation of the sample with laser light at 488 nm (CD63) and 561 nm (tubulin); *z*‐stacks are displayed as maximum intensity projections. Scale bars: 4 μm for columns 1 and 2; 1.5 μm for column 3. The data in this figure suggest that platelet size varies between individuals, but does not seem to correlate with this disease. We do not yet have a database sufficiently large to allow any conclusions to be reached as to significance.

**Figure 5:**
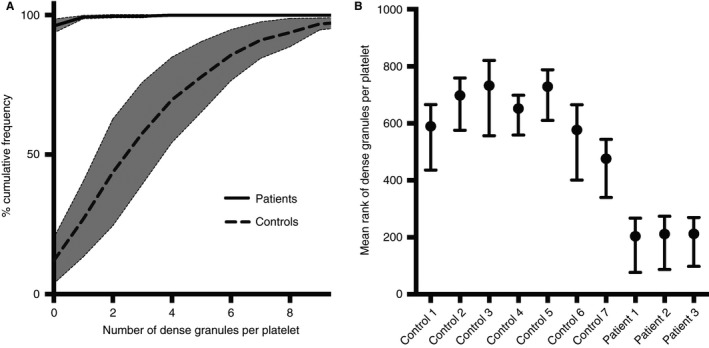
Whole mount EM analysis. Dense granules were manually counted in 1008 platelets from randomised EM images of samples from seven healthy volunteers and three patients with HPS. A cumulative frequency graph (A) reveals the spread of the data. A Kruskal‐Wallis Multiple Comparison Test (B) was used as the data had a Poisson‐like distribution and thus a non‐parametric test was needed. The mean rank of the three HPS patients was significantly different (99% confidence) from all of the controls. Mean rank is a statistic used in the non‐parametric Kruskal‐Wallis test to compare observations from different patients.

Samples of platelets taken from three patients with HPS and from seven healthy volunteers were used to test the ability of SIM (Figs. 3 and 4) to differentiate between the groups. To compare with the current reference‐standard technique, we performed a whole‐mount EM analysis (Figs. 1 and 5). Due to the stringent criteria established (Figure S1) and the use of only one analyst to blindly count all 1008 platelets, a significant difference (99% confidence) was observed as very few of the patient platelets had any dense granules at all (Fig. 5); the mean number of dense granules in the controls was 3.5 (standard deviation, 1.2), compared with 0.07 for the patients (standard deviation, 0.02). An empirical cumulative distribution function (ECDF) plot (Fig. 5A) demonstrates the clear distinction between the control and HPS patient populations. As a non‐parametric test was required to analyze significance (due to the Poisson‐like distribution of the dense granule counts), a Kruskal–Wallis test was used to compare the results (Fig. 5B) and a significant difference was seen between the controls and the patient groups (99% confidence) and between each of the patients and all of the controls, using a multiple comparison test (99% confidence). Equivalent results were obtained using anova (not shown).

Having established that our three HPS patients do indeed lack dense granules, as seen by EM, we tested the same blood samples by SRM, labeling for CD63 and tubulin and counting the number of CD63‐positive structures by SIM, using an automatic workflow that relied on no user‐defined settings and is thus entirely unbiased. We found that the mean number of CD63‐positive structures per platelet determined by SIM was 6.8 (standard deviation, 0.45) for controls, whereas for patients it was 2.4 (standard deviation, 0.48). The mean number of CD63‐positive structures for controls is about twice as many as for dense granules, as counted by EM: 3.5 as opposed to 6.8. This must reflect the presence of CD63 on other organelles within the platelets, as expected for such an itinerant membrane protein. However, despite this difference in baseline, the important finding is that (either by EM or SRM) a highly significant difference between controls and patients is seen and therefore SRM could act as an effective determinant of a dense‐granule disorder.

**Figure 6:**
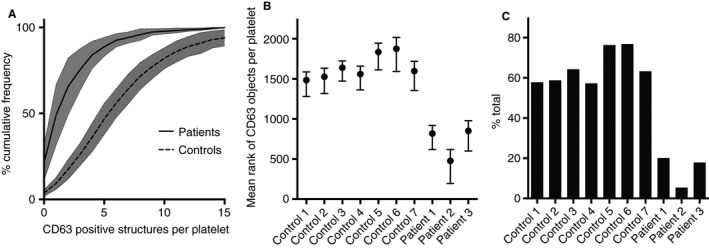
Analysis of structured illumination microscopy (SIM) data. The number of CD63 objects per platelet was counted for 2812 platelets across seven controls and three patients with Hermansky‐Pudlak syndrome (HPS) after segmentation (Fig. 2). A cumulative frequency graph (A) demonstrates the spread of the data. A Kruskal‐Wallis multiple comparison test (B) was used as the data had a Poisson‐like distribution and thus a non‐parametric test was needed. The mean rank (a statistic used in the non‐parametric Kruskal–Wallis test to compare observations from different patients) of the three HPS patients was significantly different (99% confidence) from that of all of the controls. An example readout for clinical use is shown in (C); here the data is displayed as the percentage of platelets containing 4 or more CD63 positive structures.

As a non‐parametric test was required to analyze significance (due to a Poisson‐like distribution of the CD63 data), a Kruskal–Wallis test was used to compare the results (Fig. 6B) and a significant difference was seen between the controls and the patient groups (99% confidence) and between each of the patients and all of the controls, using a multiple comparison test (99% confidence). An ECDF plot (Fig. 6A) demonstrates the clear distinction between control and HPS patient populations. In order to further simplify the ECDF readout, a threshold of four or more CD63‐positive structures per platelet was chosen and the proportion of platelets above this threshold was plotted (Fig. 6C); this clearly demonstrates the differences between controls and patients in the study and could be used as a quick read‐out to highlight a potential PGD.

**Figure 7:**
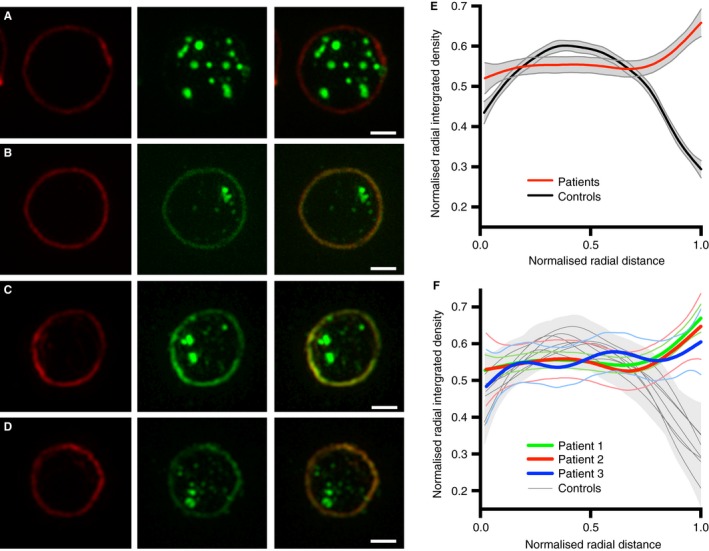
CD63 at the platelet surface in Hermansky‐Pudlak syndrome (HPS) platelets. A representative platelet is shown from the controls (A) and each of the HPS patients (B, C, D). The images show the platelet perimeter (marginal band) labelled (red) with anti‐tubulin in column 1 and the structures labelled with anti‐CD63 (green) in column 2. To highlight the change in distribution of CD63, only the green channel was contrast enhanced. Column 3 shows the merge of the green and red channels. Unlike the control (A), the three HPS patients (B, C, D) have an accumulation of CD63 at the platelet surface. To quantify this surface accumulation a radial profile algorithm was used. This calculates the normalized integrated intensity in a set of concentric rings, from the center of each platelet (0 on the *x* axis in E, F) to the platelet radius (1 on the *x* axis in E, F). The platelet radius is defined as the distance from the center of the platelet to the outer ring, where the outer ring is the maximum integrated intensity ring in the tubulin channel. The integrated intensity values are normalized for each concentric ring by division of the maximum integrated intensity ring of the platelet. Similarly, for each platelet, radial distances are normalized by division of the platelet radius. Normalization of both the radial distance and radial integrated intensity allows platelet distributions to be compared independently of the size of the platelet and the pixel intensities. A generalized additive model (GAM) was used for smoothing. As shown in E and F, only the HPS patients have an increase in signal towards the platelet surface (1 on the *x* axis in E, F). F represents the mean values of the individual controls and patients from E. Scale bars: 1 μm.

Another potential advantage of a microscopic approach is that a single image can provide data on more than one parameter, even when a single antibody is used to track one protein. One aspect of CD63 behavior in cells defective in the formation of lysosome‐related secretory organelles such as platelet granules, is that defects in the targeting of proteins to those granules can lead to an abnormal accumulation on the cell surface. This has previously been reported for cells from BLOC‐1‐deficient HPS and AP‐3‐deficient HPS 2 patients and their corresponding mouse models 26, 27. We therefore analyzed surface CD63 for these same SIM images. Using the tubulin ring as a guide for the edge of the platelets, where by definition the plasma membrane is located, we measured levels of plasma membrane‐localized CD63 in controls and patients. Quantitation (Fig. 7) shows a highly significant increase in plasma membrane‐localized CD63 in HPS patients, in line with those previous results. This preliminary finding strengthens our overall conclusions and shows how a simple sample preparation imaged by SRM can provide two independent fully quantitative measures of this particular disease.

## Discussion

The current methods for testing for a PGD typically include LTA and measurement of platelet nucleotides 4. To characterize the platelet granules, we used SRM to analyze the distribution of CD63, an itinerant integral membrane protein that traffics between post‐Golgi organelles and has been found to be present on many secretory granules, endosomes and lysosomes, as well as lysosome‐related organelles 28. Importantly, CD63 has been shown to be present in dense granules and to have an altered distribution in HPS patients 18, 29. Our data suggest that an SRM approach coupled with morphometric analyses could be an effective and rapid approach to differentiating between a patient with a platelet bleeding disorder and healthy volunteers.

The sensitivity of our analysis by SIM is demonstrated by the ability of this technique to distinguish between three HPS patients and seven controls (99% confidence), not only regarding the number of CD63‐positive structures but also the distribution of CD63 within their platelets. Indeed, we estimate, using a one‐sample *t*‐test for the two populations (patients vs. controls), that a sample size of 25 platelets would have been sufficient to give a similar probability of detection of HPS. However, because HPS causes an almost complete loss of dense granules, this is a relatively easy syndrome to diagnose. In this study, we analyzed an average of 281 platelets per person, because this greater number does not affect the time, cost or sample size required, yet would potentially increase the sensitivity of analysis to allow detection of a much less severe phenotype. It would be easy to analyze much larger numbers of platelets if this was required to further increase sensitivity.

SIM is able to resolve CD63‐positive structures and is the only technique currently reported that could potentially report morphological data from a number of alpha and dense‐granule markers at the same time, detailing quantitative information as to the distribution and aberrant localization of markers, all in one sample, which can be fixed and analyzed in parallel in large numbers.

Because conventional microscopy does not allow the resolution afforded by instruments that are not diffraction limited, SIM is becoming a more common technique (publications show an exponential rise since 2005). It is also less expensive to maintain and less labor intensive than an EM. Further, the small number of operations carried out on the platelets will improve simplicity and thus increase reliability.

Among the currently available SRM methods, SIM is the simplest to use, as samples can be easily fixed and imaged and the time‐consuming steps needed to ensure the improvement in resolution of STORM (Stochastic Optical Reconstruction Microscopy) or STED (Stimulated Emission Depletion) are unnecessary. Previous morphological dense‐granule analyses have relied upon manual counting of granules in a whole‐mount image 18, whereas the counting of CD63‐positive structures on 2812 platelets (in total) by SIM was achieved in our study in less than 5% of the time needed for the manual analysis of 1008 platelets (in total) by EM, yet gave the same result. Additionally, centrifugation of fixed platelets onto poly‐lysine‐coated coverslips reduces the amount of blood required to produce 96 coverslips of platelets to less than 1 ml, and this could potentially even be scaled down to a pinprick sample. Fixed platelets are also stable for several months at 4 °C and could be sent to a central facility for diagnosis by SIM and imaged/re‐analyzed at a later date if required, relieving local laboratories of the burden of equipment acquisition and training. Recently published FACs methods for diagnosing PGDs 8, 9 do demonstrate additional ways to carry out functional tests of platelets, but as with all functional assays, this can only be carried out immediately on site after the collection of samples, and would not detect defects in protein localization.

One other potential method that could be used to analyze dense granules would be vital staining with mepacrine. However, there are drawbacks to using this technique. Perhaps the most serious issue is that it can only be used on live platelets, making obtaining the data more difficult, and importantly precludes a real reduction in the number of laboratories carrying out the analysis. Mepacrine also renders the granules sensitive to light, so that it cannot be used in double‐ or multiple‐labelling modes in the way that the SIM approach will eventually be used. Finally, it has been reported that mepacrine labels both full and empty granules, again potentially complicating analyses.

Whilst whole‐mount EM may clearly distinguish controls from patients with HPS, the technique is difficult to standardize and is dependent on each sample being processed on the same day and counted by the same analyst to gain meaningful comparisons. The contrast within the image is often variable and can lead to difficulties in classifying dense granules. A number of sites across the North American Specialized Coagulation Laboratory Association were asked to evaluate the number of dense granules in one image of one platelet, and the number of dense granules reported ranged from six for an inexperienced site to 15–24 for experienced sites 12. Added to this is the potential for variation in sample preparation. For the purpose of our study extremely stringent criteria were used to analyze the whole‐mount EM images of 1008 platelets; the same analyst counted all images blind and on the same day. Although the result is striking (Fig. 5), the time‐consuming nature and subjective criteria required to implement this technique make it non‐ideal for clinical use. This highlights an important advantage of the SIM analysis presented here, where there is no user‐defined threshold applied to the segmentation and counting of granules. An automatic thresholding value set by ‘Moments’ in ImageJ 24 selects the parameters automatically and thus results could be easily compared between different analysts without any subjective analysis. SIM‐based methodology could easily be applied to a high‐throughput microscopy platform with automated data analysis that would present the results to a clinician for interpretation.

Current LTA methods are not specific for dense‐granule disorders and provide no information about platelet structure. A whole‐mount EM approach to analyze platelets is also limited to the visualization of dense granules and thus alpha granule disorders, such as Gray platelet syndrome, cannot be identified by this method; transmission EM of thin sections of fixed embedded platelets 30 can be used to visualize alpha granules but sample preparation to achieve this is even more time consuming and requires highly skilled technicians, and is therefore not practical to routinely implement in a clinical context.

For routine diagnostic purposes it could be argued that it is counter‐productive to categorize granules as alpha and dense granules, as there is no efficient technique to analyze both within a large number of platelets. Alpha and dense granules are distinct but many markers that are claimed to label alpha granules and dense granules do not share the same structures when analyzed by immunofluorescence. For example, Kamykowski *et al*. report that 15 different markers for alpha granules fail to show any significant co‐clustering 31. Whether a large super‐resolution morphometric dataset collected over time from many patients would reveal marker clustering associated with different diseases will therefore be of significant interest.

In 2013 it was reported that 76% of patients with HPS were initially misdiagnosed and 28% of patients had to see four to six specialists before receiving the correct diagnosis 32. Thus improvements to speed, accuracy and sensitivity would be welcome.

The procedure presented here provides a quantitative automated unbiased methodology, which can be further extended by the addition of other marker‐specific immunoprobes to simultaneously quantify other platelet structures such as α‐granules, and can also measure parameters such as the size and shape of organelles, and novel parameters that arise, such as the fraction of any membrane protein on the platelet surface, can also be extracted from the morphometric datasets to provide other or compound diagnostics. Finally, analysts would also be able to mine an increasing collection of standardized morphometric data to reveal new disease phenotypes, as well as combinations of markers that would improve future diagnosis.

## Disclosure of Conflict of Interests

D. Westmoreland and D. F. Cutler were funded by MRC Programme grant MC_UU_12018/2. J. J. Burden was funded by MRC LMCB core grant. M. Shaw, D. J. Metcalf and A. E. Knight were funded by the NMS Chemical and Biological Metrology Programme and the NPL Strategic Research Programme. W. Grimes was supported in part by the Biomedical Research Council of A*STAR (Agency for Science, Technology and Research), Singapore and partly by MRC LMCB.

## Supporting information


**Fig. S1.** Whole‐mount electron microscopy (EM) counting criteria. Whole‐mount EM of a control platelet demonstrating the counting criteria employed in this study. Only dense structures that were of a certain contrast with a defined structure and size were counted (black arrows). All counting was performed without knowledge of the origin of the platelet, in a full set of randomized images; stringent criteria were chosen, as there are many structures that resemble dense granules (white arrows) that were not counted but could affect the reliability of the results if included. Scale bars: 1 μm
