# Supplementary figures and images for: Super‐resolution microscopy as a potential approach to diagnosis of platelet granule disorders
Source: J Thromb Haemost. 2016 Mar 17;14(4):839–49. doi: 10.1111/jth.13269 (PMC4982064; doi:10.1111/jth.13269)

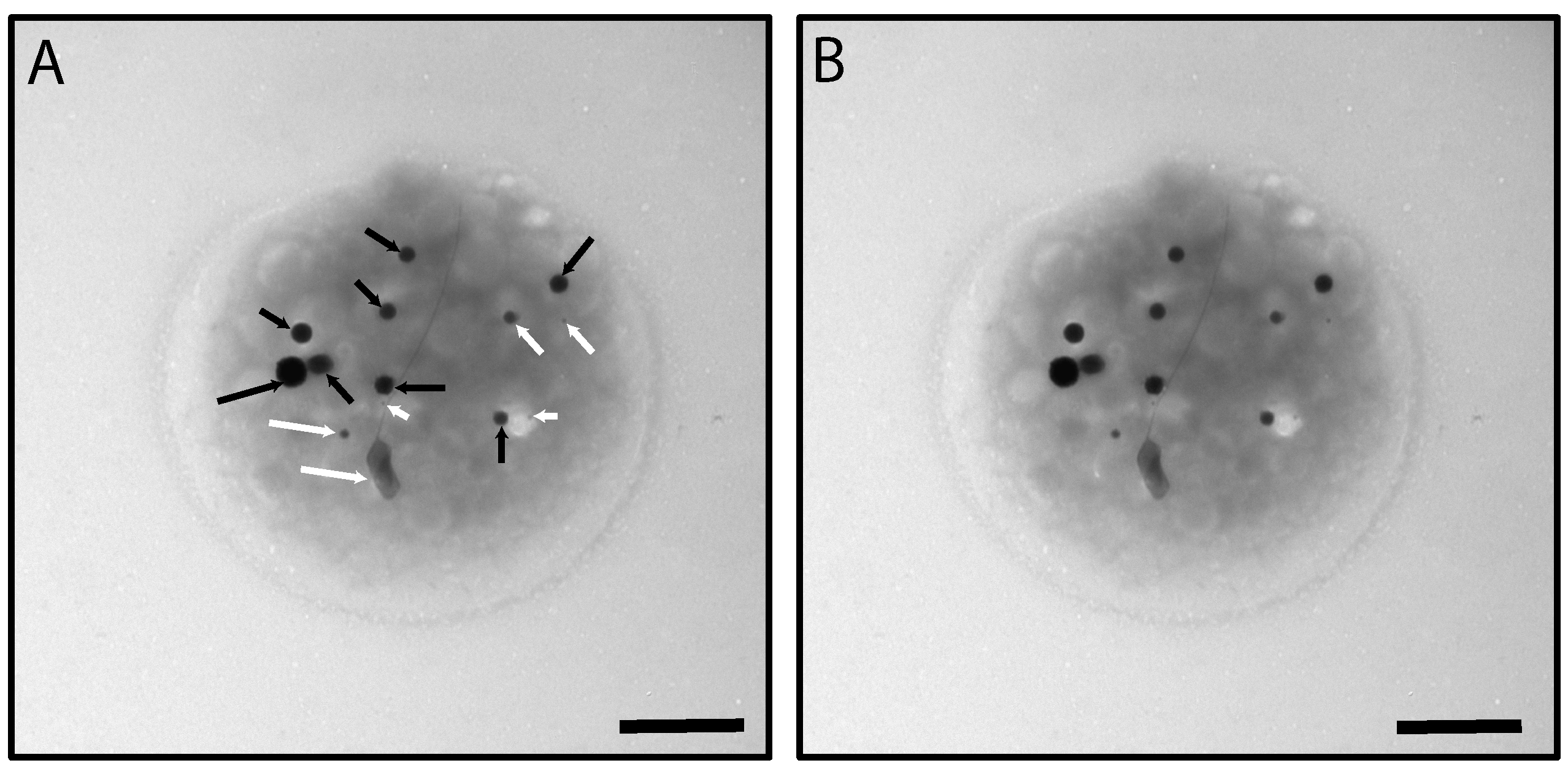

Supplement: Supplementary file 1 — Fig. S1. Whole‐mount electron microscopy (EM) counting criteria. Whole‐mount EM of a control platelet demonstrating the counting criteria employed in this study. Only dense structures that were of a certain contrast with a defined structure and size were counted (black arrows). All counting was performed without knowledge of the origin of the platelet, in a full set of randomized images; stringent criteria were chosen, as there are many structures that resemble dense granules (white arrows) that were not counted but could affect the reliability of the results if included. Scale bars: 1 μm [file JTH-14-839-s001.tif]
